# Application of quantitative lung ultrasound instead of CT for monitoring COVID-19 pneumonia in pregnant women: a single-center retrospective study

**DOI:** 10.1186/s12884-021-03728-2

**Published:** 2021-03-26

**Authors:** Qing Deng, Sheng Cao, Hao Wang, Yao Zhang, Liao Chen, Zhaohui Yang, Zhoufeng Peng, Qing Zhou

**Affiliations:** 1 Department of Ultrasound, Renmin Hospital of Wuhan University, Wuhan, 430060 Hubei China; 2 Department of Radiology, Renmin Hospital of Wuhan University, Wuhan, 430060 Hubei China

**Keywords:** Lung ultrasound, COVID-19, Pneumonia, Pregnant woman

## Abstract

**Background:**

Computed tomography (CT) is the preferred imaging technique for the evaluation of COVID-19 pneumonia. However, it is not suitable as a monitoring tool for pregnant women because of the risk of ionizing radiation damage to the fetus as well as the possible infection of others. In this study, we explored the value of bedside lung ultrasound (LUS) as an alternative to CT for the detection and monitoring of lung involvement in pregnant women with COVID-19.

**Methods:**

Clinical and LUS data of 39 pregnant women with COVID-19 were retrospectively reviewed. All LUS and CT images were analyzed to summarize the findings and calculate LUS scores and CT scores for each patient. LUS findings were compared with CT, and correlation between LUS scores and CT scores was evaluated.

**Results:**

Among the 39 pregnant women, there were 6 mild-type cases, 29 common-type cases, 4 severe-type cases, and no critical-type cases. The most common LUS findings of COVID-19 pneumonia in pregnant women were various grades of multiple B-lines (84.6%), thickened and irregular pleural lines (71.8%), pleural effusion (61.5%) and small multifocal consolidation limited to the subpleural space (35.9%). The mean LUS score at admission was 0 points in mild-type cases, 10.6 points in common-type cases and 15.3 points in severe-type cases (*P* < 0.01). The correlation between LUS scores and CT was 0.793. All patients were clinically cured and each underwent an average of three LUS follow-ups during hospitalization. The mean LUS score at discharge was 5.6 points lower than that at admission. The consistency of LUS and chest CT during follow-up was 0.652.

**Conclusions:**

Quantitative LUS scoring can effectively instead of CT for detecting and monitoring of COVID-19 pneumonia in pregnant women and protect fetuses from the risk of ionizing radiation.

## Background

The Coronavirus Disease 2019 (COVID-19) pandemic has lasted more than 1 year [1]. Unfortunately, it is unlikely that the pandemic will disappear soon. Up to now, there are more than 100,000,000 confirmed cases and more than 2,500,000 deaths have been reported worldwide, including many pregnant women [2, 3]. Severe pneumonia and acute respiratory distress syndrome are the leading causes of death in COVID-19 patients. Timely and accurate evaluation of lung lesions is very important in the treatment of patients with COVID-19. Although chest computed tomography (CT) represents the gold standard to assess the evaluate lung lesions, it is not an ideal monitoring tool for pregnant patients because of the risk of ionizing radiation to the fetus. In addition, transporting pregnant women to the radiology department for CT examination increases the risk of spread to health care providers.

Lung ultrasound (LUS) offers a convenient, inexpensive and radiation-free monitoring tool at the bedside. Many researches have highlighted the advantages of LUS in the evaluation of COVID-19 pneumonia in general population [4–6]. Whether the LUS manifestations of COVID-19 pneumonia in pregnant women are characteristic compared with the general population, and whether LUS can be used instead of CT to evaluate and monitor the lung lesions in pregnant women during COVID-19 pandemic is the focus of many obstetricians. Some studies have shown that LUS can accurately evaluate lung lesions in pregnant women with COVID-19 [7–9]. However, some clinicians suggested LUS images are non-specific and may mislead the treatment of pregnant women during COVID-19 [10, 11]. The application of LUS in pregnant women with COVID-19 remains to be further studied. In this study, we retrospectively analyzed the dynamic LUS findings of 39 pregnant women in our COVID-19 center and explored the value of LUS instead of CT for monitoring of COVID-19 pneumonia in pregnant women.

## Methods

This study was approved by the clinical research ethics committee of Renmin Hospital of Wuhan University (No. WDRY2020-K031) and was carried out in accordance with The Code of Ethics of the World Medical Association. Data were collected and analyzed to facilitate better clinical decisions and treatment.

### Study population

Thirty-nine pregnant patients who were admitted to the hospital from January 28, 2020, to April 15, 2020, were included in the study. In 29 patients, the diagnosis of COVID-19 was confirmed via real-time fluorescence reverse-transcription polymerase chain reaction (rRT–PCR), revealing positive detection of severe acute respiratory syndrome coronavirus 2 (SARS-CoV-2) nucleic acid in samples of pharynx swabs. The other 10 patients were clinically diagnosed with COVID-19. The clinical diagnosis criteria were as follows: 1. Supportive epidemiological history, including history of travel or residence in Hubei province within 2 weeks prior to the onset of illness, contact with patients from Hubei province with fever and respiratory symptoms within 14 days prior to onset, or presented with clustering onset; 2. Clinical manifestation, such as fever; normal or low levels of white blood cells or decreased lymphocyte counts at onset. 3. Imaging manifestation, whereby chest CT at an early stage shows the characteristics of multiple small patchy shadows and interstitial changes, which are more prominent in the extrapulmonary bands, and multiple ground-glass opacities and infiltrations may develop bilaterally with disease progression, with possible consolidation in severe cases.

According to the Diagnosis and Treatment Guidelines for COVID-19 (7th edition) issued by the National Health Commission of China, the severity of the disease was classified into 4 categories. Mild-type cases had mild clinical symptoms and no pulmonary changes on CT imaging. Patients with common-type disease had symptoms of fever and signs of respiratory infection with pneumonia changes on CT imaging. Severe-type cases presented with any one of the following: a. respiratory distress and respiratory rate ≥ 30/min, b. fingertip blood oxygen saturation ≤ 93% in resting conditions, or c. arterial partial pressure of oxygen (PaO_2_)/oxygen concentration (FiO_2_) ≤ 300 mmHg (1 mmHg = 0.133 kPa). Finally, critical-type cases met any one of the following criteria: a. respiratory failure requiring mechanical ventilation, b. shock, and c. ICU admission requirement due to multiple organ failure.

### Clinical characteristics

The demographics and baseline characteristics that we collected consisted of age, trimester of pregnancy, clinical signs and symptoms (such as fever, cough, shortness of breath, chest pain, fatigue, and loss of appetite), blood oxygen saturation and coexisting conditions. After thorough clinical assessment, blood samples were taken to evaluate white blood cells, neutrophils, lymphocytes, C-reactive protein and procalcitonin.

### Chest CT evaluation

The extent of lung lesions was evaluated using the CT scoring system adopted by Pan et al [12] Each of the 5 lung lobes was visually scored from 0 to 5: no involvement, 0; < 5% involvement, 1; 5–25% involvement, 2; 26–49% involvement, 3; 50–75% involvement, 4; and > 75% involvement, 5. The total CT score was the sum of the individual lobar scores (total score ranging from 0 to 25).

### Bedside LUS examination

The machine used was a GE Vivid™ iq ultrasonography (GE Healthcare, China) equipped with a convex C1–5-RS probe. The frequency was set at 3.5 MHz, the depth was set at 10 cm, and the gain was adjusted to obtain the best possible image. The bedside lung ultrasound examination was performed by a sonographer with 4 years’ experience in LUS. According to the recommendations of Dargent A et al. [13], the complete 12-zone LUS examination was performed with patients in the supine or near-to-supine position. The chest wall was divided into 12 zones: 2 anterior zones, 2 lateral zones and 2 posterior zones per side. The superior and inferior zones were divided by the third intercostal space. Videoclips were recorded throughout the respiratory cycle for subsequent off-line analysis. The doctors were blinded to the clinical data when analyzing the images.

**Fig. 1:**
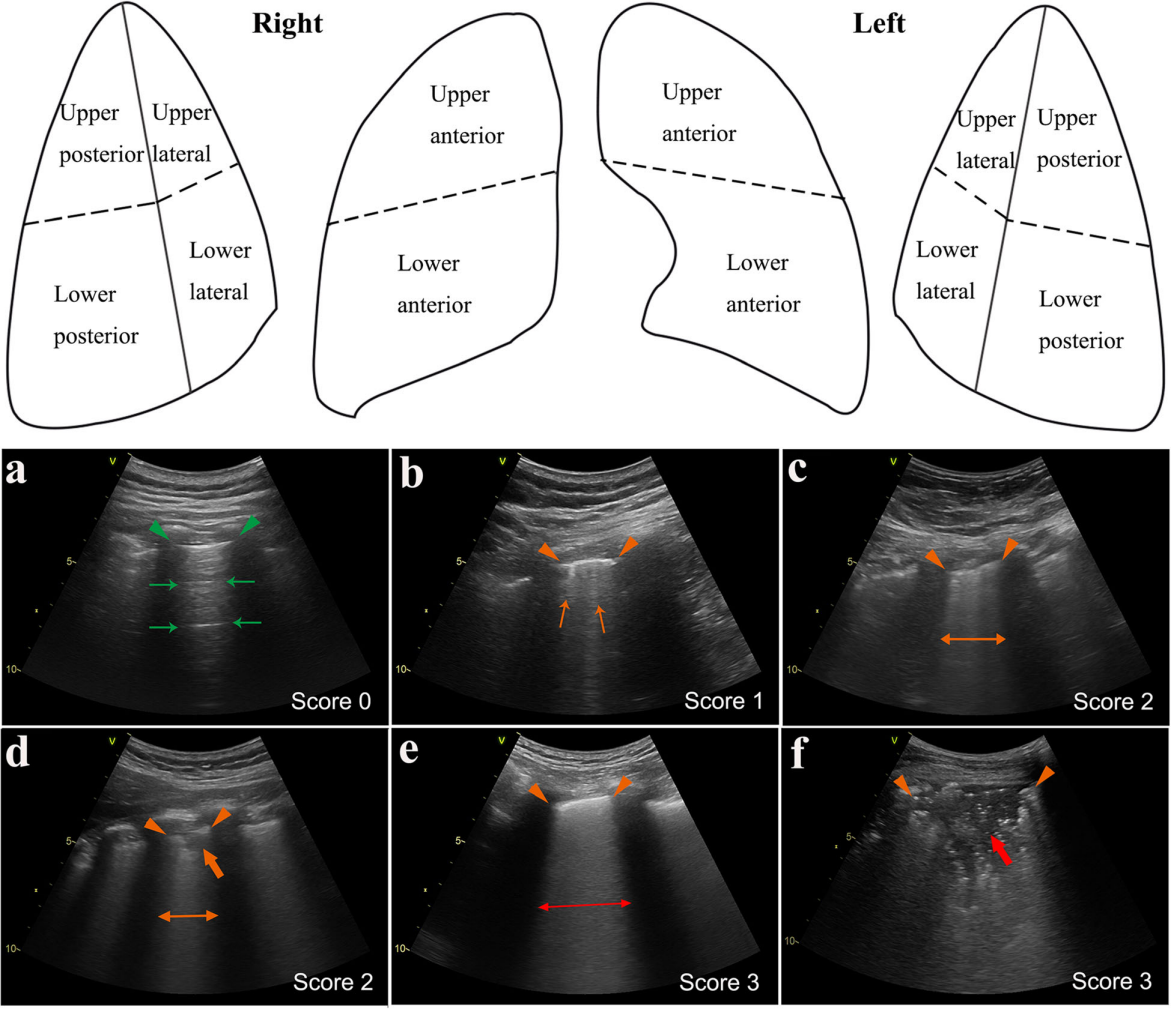
Schematic diagram of the lung ultrasound protocol and the scoring system. Each lung was separated into six quadrants: anterior, lateral and posterior zones were separated by the anterior and posterior axillary lines, with each zone divided into upper and lower portions by the third intercostal space. **a** A normal lung pattern was identified by the presence of a clear pleural line (indicated by green triangular arrowheads) and A-lines (marked with green thin arrows), scored as 0; **b** a small number of B-lines (marked with orange thin arrows), scored as 1; **c-d** the presence of crowded B-lines (indicated by orange thin double arrow) or consolidations limited to the subpleural space (marked with orange thick arrow), scored as 2; **e-f** the presence of confluent B-lines (indicated by red thin double arrow) or mass consolidation with dynamic air bronchograms (marked with red thick arrow), scored as 3. Thickened, irregular and interrupted pleural lines are indicated by orange triangular arrowheads

Each zone was scored according to the LUS pattern as follows [6]: a normal lung pattern was identified by the presence of normal lung sliding with A-lines or fewer than two isolated B-lines and was scored as 0; the presence of 3 or more well-spaced B-lines presented in a single intercostal space was scored as 1; the presence of crowded B-lines with or without consolidation limited to the subpleural space was scored as 2; and the presence of confluent B-lines or a tissue pattern characterized by dynamic air bronchograms that was defined as lung consolidation was scored as 3. The schematic diagram is displayed in Fig. 1. The most severe ultrasound finding can be considered representative of the entire zone, and the most severe ultrasound findings observed in each zone were recorded. For each lung zone, a 0- to 3-point score was given (total score ranging from 0 to 36).

To dynamically assess lung lesions, follow-up of bedside LUS was performed on all patients. Patients with LUS scores that had increased ≥2 points compared with previous examination were defined as having disease progression. Similarly, a reduction of ≥2 points in LUS score compared with the previous examination was defined as improvement. Changes in LUS score within 1 point were defined as no change.

### Inter- and intra-observer variability of LUS

The lung ultrasound images were analyzed and scored by 2 doctors with 3 years’ experience in LUS. Two doctors were blinded to each other and the clinical data. Random ultrasound images of 20 examinations were analyzed by two independent observers to assess inter- and intra-observer variability. The two observers were blinded to each other and neither of them were participated in the lung ultrasound examination. The interval between two analyses performed by the same observer was more than a month. Inter- and intra-observer consistency were defined as the same images acquired the same lung ultrasound score.

### Statistical analysis

Continuous numeric variables are expressed as the mean value ± standard deviation, and dichotomous variables are expressed as the frequency number (%) or median [interquartile range]. Variables were compared among three groups using the Chi-square test. Correlations between LUS scores and CT scores were analyzed using the Pearson coefficient if the data is normally distributed. A Kappa consistency check was applied to evaluate the consistency of LUS and CT. Statistical significance was defined at a level of P<0.05. All statistical tests were analyzed with SPSS software (version 20.0, SPSS Inc., Chicago, USA).

## Results

### Demographic and clinical characteristics

The demographic and clinical characteristics are shown in Table 1. The median age was 30 years (IQR: 27–32; range: 24–40 years). Of the 39 patients, 27 (69.2%) were in the third trimester of pregnancy, 8 (20.5%) were in the second trimester, and 4 (10.3%) were in early pregnancy. Regarding clinical manifestations, fever (46.2%), cough (33.3%), diarrhea (18.0%) and fatigue (18.0%) were the most prevalent. There were 16 patients without obvious clinical symptoms. Of the 39 patients, there were 6 mild-type cases, 29 common-type cases and 4 severe-type cases. Nine patients (23.0%) presented with pregnancy complications, including 3 with gestational hypertension (7.7%), 2 with chronic nephritis (5.1%), 2 with gestational diabetes mellitus (5.1%), and 2 with thrombocytopenia (5.1%). During hospitalization, 21 (53.9%) and 18 (46.2%) patients exhibited a reduction in lymphocytes and elevated levels of C-reactive protein, respectively. Three severe-type cases had decreased oxygen saturation, whereas oxygen saturation was normal in the other cases.

**Table 1:** Clinical characteristics of pregnant women with COVID-19

Patient information	All Patients (*n* = 39)	Disease type			
		Mild (*n* = 6)	Common (*n* = 29)	Severe (*n* = 4)	*P* value
Age, years	30 (27–32)	29 (27–31)	30 (27–32)	30 (28–31)	0.867
Early trimester	4 (10.3%)	1 (16.7%)	3 (10.4%)	0	0.711
Second trimester	8 (20.5%)	0	7 (24.1%)	1 (25.0%)	0.431
Third trimester	27 (69.2%)	5 (83.3%)	19 (65.5%)	3 (75.0%)	0.750
Time from illness onset to hospital admission, days	9 (7–11)	8 (6–11)	9 (7–11)	11	0.846
**Signs and symptoms**					
Fever, n (%)	18 (46.2%)	1 (16.7%)	13 (44.8%)	4 (100.0%)	0.027
Cough, n (%)	13 (33.3%)	1 (16.7%)	8 (27.6%)	4 (100.0%)	0.015
Diarrhea, n (%)	7 (18.0%)	1 (16.7%)	4 (13.8%)	2 (50.0%)	0.196
Sore throat, n (%)	6 (15.4%)	0	4 (13.8%)	2 (50.0%)	0.139
Fatigue, n (%)	7 (18.0%)	0	4 (13.8%)	3 (75.0%)	0.023
Shortness of breath, n (%)	5 (12.8%)	0	2 (6.9%)	3 (75.0%)	0.005
Chest pain/tightness, n (%)	4 (10.3%)	0	1 (3.5%)	3 (75.0%)	0.004
No signs or symptoms	16 (41.0%)	4 (66.7%)	12 (41.4%)	0	0.134
**Pregnancy complications**					
Gestational hypertension, n (%)	3 (7.7%)	1 (16.7%)	2 (6.9%)	0	0.600
Chronic nephritis, n (%)	2 (5.1%)	0	2 (6.9%)	1 (25.0%)	0.334
Gestational diabetes mellitus, n (%)	2 (5.1%)	0	2 (6.9%)	1 (25.0%)	0.334
Thrombocytopenia, n (%)	2 (5.1%)	0	2 (6.9%)	0	1.000
**Laboratory tests**					
Leucocytes increased (> 9.5 × 10^9^/L)	12 (30.8%)	1 (16.7%)	8 (27.6%)	3 (75.0%)	0.150
Neutrophils increased (> 6.3 × 10^9^/L)	17 (43.6%)	2 (33.3%)	12 (41.4%)	3 (75.0%)	0.561
Lymphocytes decreased (< 1.1 × 10^9^/L)	21 (53.9%)	1 (16.7%)	16 (55.2%)	4 (100.0%)	0.046
C-reactive protein increased (> 5.0 mg/L)	18 (46.2%)	2 (33.3%)	13 (44.8%)	3 (75.0%)	0.442
Procalcitonin level increased (> 0.1 μg/L)	12 (30.8%)	1 (16.7%)	9 (31.0%)	2 (50.0%)	0.529
Oxyhemoglobin saturation, %	96 (95–98)	98 (97–98)	96 (95–97)	92 (91–93)	0.001
Decreased oxygen saturation (≤ 93%)	3 (7.7%)	0	0	3 (75.0%)	0.000

Each value represents the median (interquartile range) or the number (%)

### Clinical outcome

Twenty-six patients were given low flow oxygen therapy. None of the patients used mechanical ventilation or were sent to the ICU. Fifteen patients had normally progressing pregnancies. One patient had a spontaneous abortion at 6 weeks of gestation. Twenty-three patients gave birth during their hospitalization. Among the 23 puerperae, 1 had a natural birth; the other children were all delivered by cesarean section. Except for one neonate who had an Apgar score of 7 (1 ~ 5 min), the Apgar scores of the other neonates were 9 ~ 10 (1 ~ 5 min). The nucleic acid tests of all the children were negative. As of April 25th, 2020, all patients were cured and discharged from the hospital; no cases deteriorated into the critical type, and there were no deaths. The discharge criteria were as follows: (1) body temperature returned to normal for more than 3 days; (2) respiratory symptoms improved obviously; (3) pulmonary imaging showed obvious absorption of inflammation; and (4) nucleic acid tests were negative twice consecutively using respiratory tract samples such as sputum and nasopharyngeal swabs (sampling interval being at least 24 h). The average hospitalization time was 14 days (IQR: 9–23 days; range: 5–41 days).

### Chest CT characters and scores

All patients underwent CT examination before admission. The median interval from CT examination to hospital admission was 1.5 days (range: 0 ~ 3 days). The CT characteristics and lesion distribution before admission are summarized in Table 2. CT examination revealed no abnormalities in the 6 mild-type cases, and the other 30 patients had lung lesions. The most frequent CT findings were ground-glass opacity (29/39), followed by pleural effusion (19/39) and consolidation (16/39). Among the 33 patients with positive lung CT findings, 26 (76.9%) cases had bilateral and multifocal involvement and 31 cases (93.9%) had peripheral involvement. In common-type cases, an average of 2.9 lobes were involved; in severe-type cases, 4.8 lobes were involved. The mean CT score was 6.6 points in common-type cases and 11.7 points in severe-type cases.

**Table 2:** Lung CT of pregnant women before admission

	All Patients (*n* = 39)	Disease type			
		Mild (*n* = 6)	Common (*n* = 29)	Severe (*n* = 4)	*P* value
**Lung CT findings**					
Normal	6 (15.4%)	6 (100.0%)	0	0	0.000
Ground glass opacification	29 (74.4%)	0	25 (86.2%)	4 (100.0%)	0.000
Consolidation	16 (41.0%)	0	12 (41.4%)	4 (100.0%)	0.004
Crazy-paving pattern	11 (28.2%)	0	8 (27.6%)	3 (75.0%)	0.031
Pleural effusions	19 (48.7%)	0	15 (51.7%)	4 (100.0%)	0.003
**Lesion distribution found by CT**					
1 lobe involved	3 (7.7%)	0	3 (10.4%)	0	1.000
2 lobe involved	6 (15.4%)	0	6 (20.7%)	0	0.607
3 lobe involved	11 (28.2%)	0	11 (37.9%)	0	0.100
4 lobe involved	9 (23.1%)	0	8 (27.6%)	1 (25.0%)	0.457
5 lobe involved	4 (10.3%)	0	1 (3.5%)	3 (75.0%)	0.004
Periphery involvement	31 (79.5%)	0	27 (93.1%)	4 (100.0%)	0.000
Bilateral involvement	26 (66.7%)	0	22 (75.9%)	4 (100.0%)	0.000
**Lung CT score**	6.1 ± 4.8	0	6.6 ± 3.5	11.7 ± 2.7	0.000

During hospitalization, all pregnant women refused to have CT follow-up before delivery. After delivery, 23 patients underwent repeat CT examination. Compared with the condition before admission, CT follow-up showed that the lung lesions had improved in 20 patients and had become aggravated in 3 patients.

### Bedside LUS findings and scores

The first LUS was performed within 24 h of admission. The LUS acquired positive findings in 34 patients. The most common LUS findings of COVID-19 pneumonia in pregnant women were increased B-lines to different degrees (33/39), thickening and irregularity of the pleural line (28/39), pleural effusions (24/39), and small multifocal consolidation limited to the subpleural space (14/39). Mass consolidations with dynamic air bronchograms were not found in this study. Most patients showed involvement of 5 ~ 6 zones. The mean LUS score was 0 points in mild-type cases, 10.6 points in common-type cases and 15.3 points in severe-type cases. The LUS findings and scores are displayed in Table 3.

**Table 3:** Lung ultrasound of pregnant women on admission

	All Patients (*n* = 39)	Disease type			
		Mild (*n* = 6)	Common (*n* = 29)	Severe (*n* = 4)	*P* value
**Lung ultrasound findings**					
Normal	5 (12.8%)	5 (83.3%)	0	0	0.000
Thickening and irregularity of pleural lines	28 (71.8%)	1 (16.7%)	23 (79.3%)	4 (100.0%)	0.009
Increased B-lines	33 (84.6%)	1 (16.7%)	28 (96.6%)	4 (100.0%)	0.000
Confluent B-lines	21 (53.9%)	0	17 (58.6%)	4 (100.0%)	0.002
Small consolidations limited to the subpleural space	14 (35.9%)	0	11 (37.9%)	3 (75.0%)	0.049
Mass consolidations characterized by dynamic air bronchograms	0	0	0	0	1.000
Pleural effusions (The maximum depth > 1 cm)	22 (56.4%)	0	18 (69.2%)	4 (100.0%)	0.002
**Involved zones detected by ultrasound**					
1–2 zones	4 (10.3%)	1 (16.7%)	3 (10.4%)	0	0.711
3–4 zones	4 (10.3%)	0	4 (13.8%)	0	1.000
5–6 zones	14 (35.9%)	0	14 (48.3%)	0	0.027
7–8 zones	7 (18.0%)	0	7 (24.1%)	0	0.390
9–10 zones	2 (5.1%)	0	1 (3.5%)	1 (25.0%)	0.217
11–12 zones	3 (7.7%)	0	0	3 (75.0%)	0.000
**Lung ultrasound score**	9.0 ± 6.4	0	10.6 ± 3.8	15.3 ± 2.1	<0.01

**Fig. 2:**
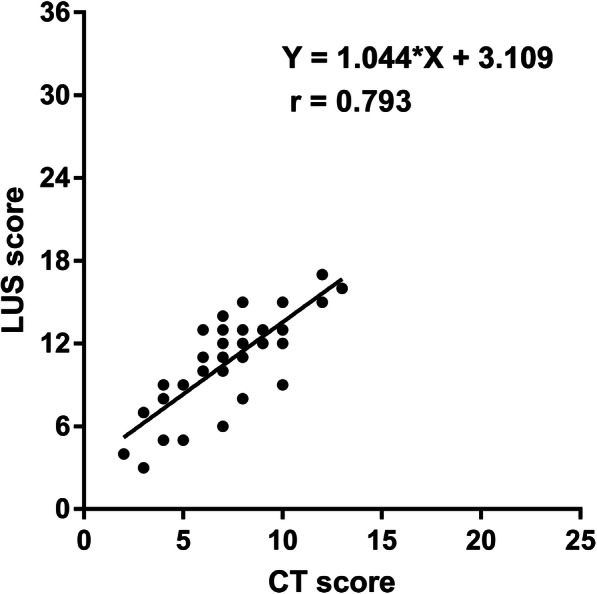
The correlation between lung ultrasound scores and CT scores in pregnant patients

**Fig. 3:**
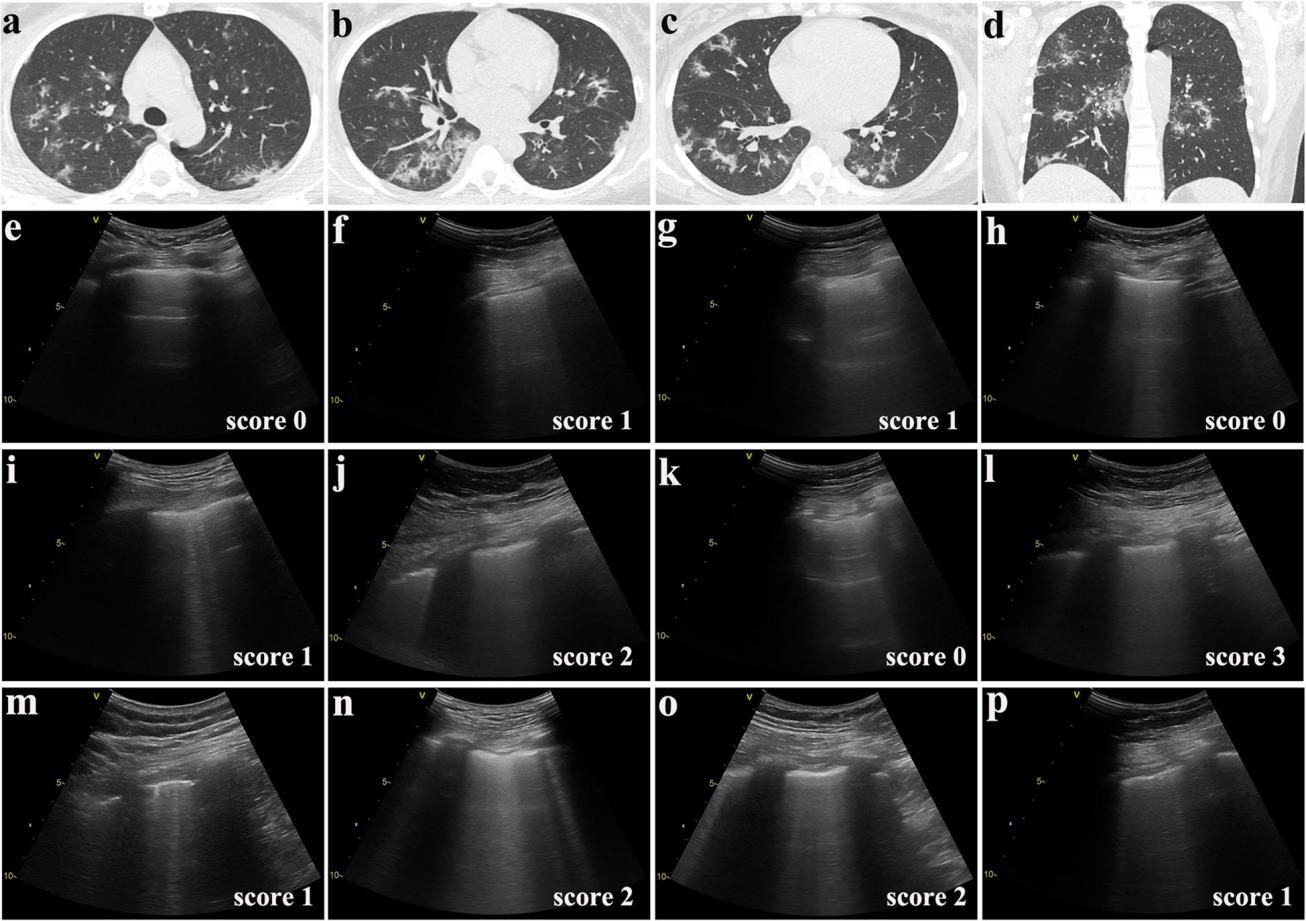
Chest CT and lung ultrasound imaging of a typical patient. **a-d** Chest CT showed involvement of all five lobes of the bilateral lungs in this patient, and the total CT score was 10 points. **e-p** The lung ultrasound findings in 12 zones. **e**: right anterior upper zone, **f**: right anterior lower zone, **g**: left anterior upper zone, **h**: left anterior lower zone, **i**: right lateral upper zone, **j**: right lateral lower zone, **k**: left lateral upper zone, **l**: left lateral lower zone, **m**: right posterior upper zone, **n**: right posterior lower zone, **o**: left posterior upper zone, **p**: left posterior lower zone. The total ultrasound score was 14 points

The data of LUS scores and CT scores on admission is normally distributed. Pearson correlation analysis revealed a high correlation between LUS scores and CT scores on admission (*r* = 0.793, *p* < 0.01), as shown in Fig. 2. A typical case is displayed in Fig. 3.

### LUS in follow-up

Bedside LUS follow-up was performed for all patients. In our study, all patients refused to have chest X-ray films or CT follow-up before delivery, and LUS was the only accepted imaging examination. Each patient underwent 1 ~ 6 LUS examinations, with a median of 3 times. The LUS scores of all patients at discharge were significantly improved compared with those at admission, with an average decrease of 5.6 points. Twenty-three patients continued to improve after admission; the other 16 patients first worsened and then improved. The results of the LUS follow-up are shown in Table 4.

**Table 4:** Lung ultrasound follow-up of pregnant patients

	Lung ultrasound follow-up					
	First	Second	Third	Fourth	Fifth	Sixth
Time interval since the last lung ultrasound examination (Days)	3 (2 ~ 5)	3 (2 ~ 5)	4 (2 ~ 7)	6 (3 ~ 9)	7 (3 ~ 11)	12
Patients underwent follow-up	39	36	21	13	4	1
Lesion progression	16	8	3	0	0	0
No change	14	8	5	3	0	0
Lesion improvement	9	20	13	10	4	1
Patient with pleural effusions	26	16	2	0	0	0

**Fig. 4:**
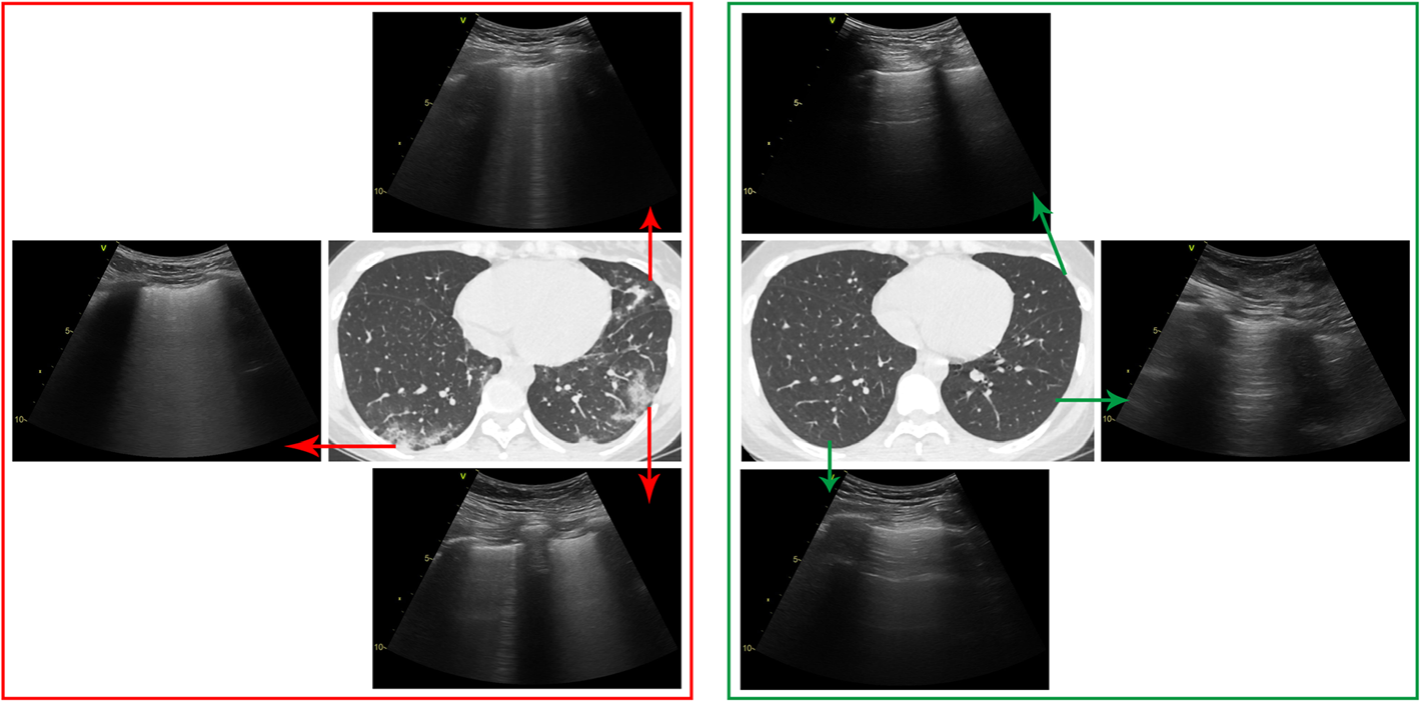
Lung ultrasound follow-up of a pregnant patient. The red frame on the left shows the lung images of the patient on admission; both ultrasound and CT showed obvious lesions in the peripheral zone. After 34 days of treatment, the lesions were completely absorbed at discharge, and both ultrasound and CT images were normal, as shown in the green frame on the right

Twenty-three patients with late pregnancy received both lung ultrasound and CT follow-up after delivery. The follow-up results were divided into three types: progression, no change and improvement. According to the Kappa test, the consistency of LUS and CT in the follow-up of lung lesions was 0.652 (*p* < 0.01). The LUS and CT follow-up of a pregnant woman who returned to normal at discharge were shown in Fig. 4.

### Inter- and intra-observer variability of LUS scores

The LUS scores between the two observers showed good consistency, with an ICC of 0.904 (95% confidence interval 0.872–0.937). The intra-observer consistency of LUS scores is also well, with an ICC of 0.960 (95% confidence interval 0.933–0.985).

## Discussion

The LUS manifestations of COVID-19 in the general population have been reported in many studies [5, 6, 14, 15]. The value of LUS in pregnant women with COVID-19 still needs to be further explored. In this study, we used an LUS score to evaluate and follow-up COVID-19 pneumonia in a group of pregnant women. Our study confirmed that the most common LUS findings of COVID-19 pneumonia in pregnant women were various grades of multiple B-lines (84.6%), thickened and irregular pleural lines (71.8%), pleural effusion (61.5%) and small multifocal consolidation limited to the subpleural space (35.9%). Compared to the general population, the LUS findings in pregnant women have certain characteristics: relatively mild sonographic manifestations and a high incidence of pleural effusion. Quantitative LUS scores correlated highly with chest CT findings and could effectively evaluate lung lesions in pregnant women. LUS is a safe, accurate and quantifiable monitoring tool for pregnant women with COVID-19 pneumonia.

Lungs are the main target organs affected by SARS-CoV-2, and severe respiratory distress caused by lung damage is the leading cause of death in patients with COVID-19. Thus, accurate evaluation of lung damage is crucial for disease treatment and control. The SARS-CoV-2 nucleic acid test is the diagnostic criterion for COVID-19. However, the nucleic acid test is affected by the collection of respiratory samples and lacks sufficient sensitivity and stability, with variable sensitivities from 37 to 71%. In addition, the nucleic acid test cannot reflect the severity of COVID-19. Conversely, chest CT can rapidly and accurately evaluate lung injury in patients and guide clinical management and is considered the gold standard of lung imaging. Nonetheless, CT is not suitable for repeated use in some special groups, such as pregnant women and children. As a vulnerable population in the COVID-19 pandemic, pregnant women and children should be treated cautiously, and a safer lung imaging technique is needed. LUS is a novel method for detecting certain lung diseases. Compared with CT, LUS has the advantages of being simple, convenient, radiation free, and repeatable. It may meet the special needs of pregnant women and children with COVID-19 pneumonia [8, 16].

Our study showed that the main manifestations of LUS in pregnant patients with COVID-19 included thickening and irregularity of the pleural line, an increase in B lines, multifocal small consolidation limited to the subpleural space and a small amount of pleural effusion, similar to those of the general population [14]. In addition, LUS in pregnant women showed some characteristics that differ from those of the general population, mainly reflected in the following two aspects. First, the lung involvement of pregnant women with COVID-19 was significantly less severe than that of the general population. In our study, most cases were the mild or common type, with only four severe-type cases and no critical-type cases. Severe sonographic findings, such as mass consolidations with dynamic air bronchograms or pneumothorax, did not appear in our cohort. Second, the incidence of pleural effusion in pregnant women was significantly higher than that in the general population. Previous study has shown that the incidence of pleural effusion in the general population with COVID-19 is approximately 3.4–8.7% and the appearance of pleural effusion indicated that the patient was critically ill [17], but in our study, the incidence of pleural effusion in pregnant women with COVID-19 was as high as 61.5% and all pregnant women have a good clinical outcome. Similar results were also reported in the study of Gong et al. [18]. In their study, chest CT scan showed that six cases in ten pregnant women with COVID-19 pneumonia (60%) had small bilateral pleural effusion, which was not in line with the previous reports that pleural effusion is rare in general population with COVID-19. The high incidence of pleural effusion in pregnant women may be related to SARS-CoV-2 infection or more likely to physiological changes during pregnancy. First, the levels of C-reactive protein and procalcitonin were elevated in 46.2 and 30.8% patients in our study, which indicating certain inflammatory reactions. Inflammation may cause congestion of the visceral pleura and increased vascular permeability, resulting in pleural effusion. Second, pregnant women are prone to chest leakage due to physiological increase of blood volume and decrease of plasma colloid osmotic pressure. Our study confirmed that the high incidence of pleural effusion and good clinical outcome are the characteristics of pregnant women. It is significant different from the general population.

Because ultrasound imaging of COVID-19 pneumonia has good inter- and intra-observer consistency and can be easily understood by clinicians, LUS is being used in many departments, including in obstetrics department [19]. However, how to quantify LUS findings for global assessment of the severity of pulmonary lesions, there is still lack of a unified standard. In this study, we divided the lungs into 12 zones and used a quantitative ultrasound score to represent the involvement of the lungs. It was interesting that there was a good linear correlation between the LUS scores based on the 12-zone protocol and the CT scores based on the 5-lung-lobe protocol. Although LUS results are related to the degree of aeration of the lung’s outer and subpleural layers, they can effectively reflect the condition of lung involvement. Previous study has shown that most COVID-19 pneumonia cases present with peripulmonary and subpleural involvement in the early stage [20], and this pathological feature is easy to detect by ultrasound. These characteristics of COVID-19 pneumonia provide an ideal application condition for LUS.

Furthermore, LUS has a significant advantage over gold-standard chest CT for convenient follow-up. Ultrasound involves no radiation, and the examination can be performed anytime and anywhere. In our study, all pregnant women refused repeat CT examination before delivery because of concerns about the potential risk of ionizing radiation to the fetus. However, they all accepted LUS follow-up. In our study, an average of three LUS follow-ups were performed for each pregnant woman. We found higher initial LUS scores were associated with longer hospital stays and more follow-ups. All patients with an initial LUS score greater than 13 points had a hospital stay of more than 20 days. The LUS scores of all patients at discharge were significantly improved compared with those at admission, with an average decrease of 5.6 points. The results of LUS follow-up were consistent with the patient’s clinical outcome and CT. Of note, except for 23 postpartum women who received CT re-examination, the other 16 pregnant women chose LUS as the only imaging follow-up during their hospitalization. For these 16 pregnant women, the criteria for their discharge were defined as a significant improvement in clinical symptoms, two consecutive negative nucleic acid tests, and a significant reduction in LUS score. Our study indicates that LUS plays a useful role in the evaluation of the patient’s treatment and prognosis.

It must be acknowledged that our research has some limitations. First, LUS is a surface imaging technique, it owes its accuracy to the fact that nearly all lung pathologies relevant to COVID-19 have peripheral involvement. Even a systematic LUS examination can only show approximately 70% of the lung surface, and lesions located in the blind area of ultrasound would be missed. Second, COVID-19 is a highly contagious disease, and its treatment has been coordinated by the government. As a special group, pregnant women received more attention and were given priority for treatment. They were diagnosed earlier and treated actively. In this research, except for four severe-type cases, the other pregnant patients all had mild-type and common-type disease. There were no critical-type patients in our sample. Therefore, our study may not be representative of a more seriously ill patient population.

## Conclusions

In conclusion, lung ultrasound findings in pregnant women with COVID-19 have certain characteristics. The scoring system provides a quantitative tool for LUS findings. LUS scores correlated well with CT findings and effectively reflected the severity of lung lesions. Our study shows that quantitative LUS can be considered a reliable follow-up tool for dynamic lung monitoring in pregnant women with COVID-19. It can reduce the use of chest CT and protect pregnant women and fetuses from the risk of radiation damage.

## Data Availability

The data that support the findings of this study are available from the corresponding author but restrictions apply to the availability of these data, which were used under license for the current study, and so are not publicly available. Data and materials in the current study are available from the corresponding author upon reasonable request and permission.

## Abbreviations

SARS-CoV-2: Severe Acute Respiratory Syndrome Coronavirus 2
COVID-19: Corona Virus Disease 2019
CT: Computed Tomography
LUS: Lung Ultrasound
